# Removal of an intra-abdominal desmoplastic small round cell tumor by repetitive debulking surgery: A case report and literature review

**DOI:** 10.3892/ol.2014.1913

**Published:** 2014-02-26

**Authors:** JIRO SHIMAZAKI, GYO MOTOHASHI, KIYOTAKA NISHIDA, TAKANOBU TABUCHI, HIDEYUKI UBUKATA, TAKAFUMI TABUCHI

**Affiliations:** Department of Gastrointestinal Surgery, Ibaraki Medical Center, Tokyo Medical University, Ami, Ibaraki 300-0395, Japan

**Keywords:** desmoplastic small round cell tumor, prognosis, debulking surgery

## Abstract

In the current study, a case of recurrent desmoplastic small round cell tumor (DSRCT) is presented, which was successfully treated by repetitive debulking surgery. In May 2010, a 39-year-old male, with a history of surgical resection of intra-abdominal DSRCT, visited the Ibaraki Medical Center, Tokyo Medical University Hospital (Ami, Japan) with severe lower abdominal discomfort. Abdominal computed tomography revealed a large tumor in the pouch of Douglas with a small number of nodules in the abdominal cavity. The recurrent DSRCT was diagnosed and removed via lower anterior resection; however, complete resection was impossible due to multiple peritoneal metastases. One year later, the patient developed pain in the right groin due to the growth of metastasized tumor cells in the groin lymph nodes. The affected lymph nodes were removed utilizing an extra-peritoneal approach. At the time of writing, the patient continues to survive without any symptoms 60 months since the initial surgery. In conclusion, surgical debulking is a significant procedure for relieving patient symptoms as well as improving the survival time of patients with metastatic and recurrent DSRCT.

## Introduction

Desmoplastic small round cell tumor (DSRCT), which was first described by Gerald and Rosai (1), is a rare, high grade and aggressive malignant tumor, defined as a mesenchymal neoplasm that grows along serosal surfaces. DSRCT often affects the abdominal and/or pelvic peritoneum, predominantly affecting young adult males (2). Despite aggressive combination interventions, such as poly-chemotherapy, debulking surgery and whole abdominal radiation, the therapeutic management of DSRCT remains unsatisfactory and subsequently has a poor patient prognosis (3). Surgical resection is only recommended for non-metastatic disease with combination chemoradiotherapy as an adjunct, however, the outcome is often unefficacious, predominantly due to disease recurrence. For patients in the advanced stages of the disease, symptom control is crucial as the aforementioned interventions only marginally impact survival, thus, palliation of secondary symptoms is of paramount importance (4). Furthermore, it is essential to decide on appropriate subsequent treatments as currently, there is no consensus on any such strategy. Therefore, in the present study, a patient with recurrent and metastatic DSRCT is described, who received surgical resection in combination with chemotherapy, but was successfully treated by the surgery alone. Patient provided written informed consent.

## Case report

**Figure 1:**
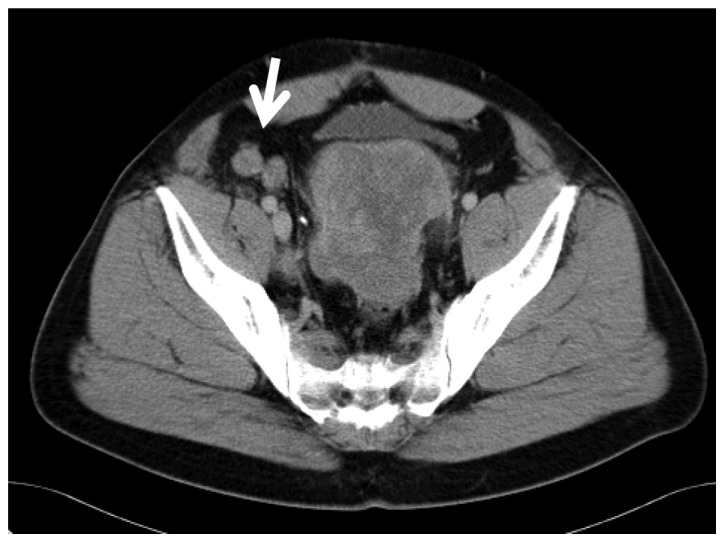
Abdominal enhanced computed tomography of the pelvis demonstrating a large mass with numerous nodules (arrow).

**Figure 2:**
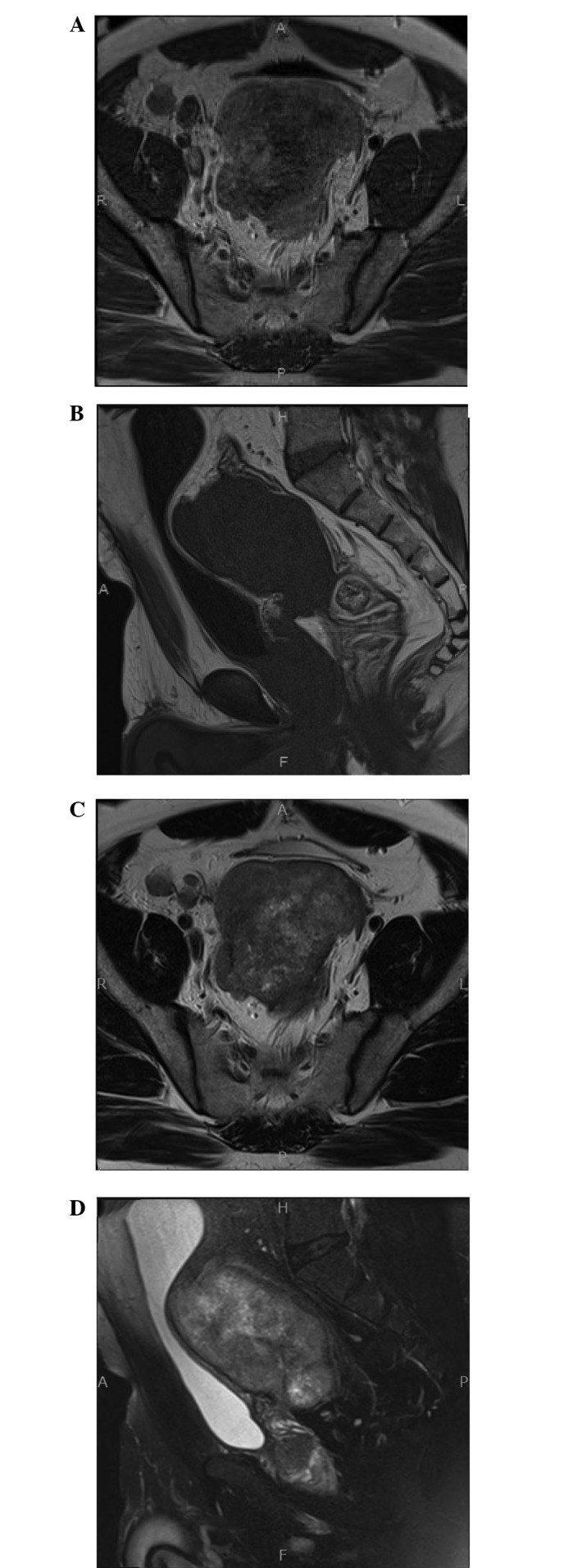
Magnetic resonance imaging. demonstrates a large lobulated mass, which compressed the bladder and rectum. (A) Axial T1-weighted. (B) Sagittal T1-weighted. (C) Axial T2-weighted. (D) Sagittal T2-weighted.

In May 2010, 39-year-old Iranian male visited the Ibaraki Medical Center, Tokyo Medical University Hospital (Ami, Japan) presenting with lower abdominal and pelvic pain together with constipation. His medical records revealed that he had undergone palliative surgery at another hospital for a large intra-abdominal tumor two years previously. Histological diagnosis of the resected specimen indicated DSRCT. One year after the first surgery, the residual tumor had grown rapidly and he had received anticancer medications. However, the residual tumor appeared to be worsening and the patient complained of debilitating symptoms. Physical examinations on the individuals’ admission to the Ibaraki Medical Center, Tokyo Medical University Hospital revealed lower abdominal tenderness without a palpable mass. The majority of the laboratory assessment results were normal, with the exception of a slight liver function disorder. Furthermore, numerous tumorigenic factors, including carcinoembryonic antigen, carbohydrate antigen (CA) 125, CA 19-9 and neuron specific enolase (NSE) were within the normal range. Abdominal enhanced computed tomography (CT) revealed a large mass (size, 18×7 cm) with slight heterogeneous enhanced areas in the pouch of Douglas, coupled with nodules in the abdominal cavity (Fig. 1). In magnetic resonance imaging (MRI), the large mass in the pouch of Douglas appeared hypointense in the T1-weighted images and heterogeneous hyperintense in the T2-weighted images (Fig. 2). In addition, the mass had compressed the rectosigmoid colon and the bladder. The diagnosis was, therefore, metastasis and recurrence of a DSRCT that was growing rapidly.

Among the several treatment strategies available, the patient opted for surgical excision of the tumor. A laparotomy was performed to relieve the patient’s symptoms, during which a voluminous mass was identified that had occupied the pouch of Douglas. Furthermore, numerous small nodules were observed on the omentum, which were coupled with diffuse peritoneal seeding on the surface of the diaphragm. The large mass had penetrated the anterior wall of the rectum, however, the bladder and the urethra were not affected. The tumor was removed via a low anterior resection and a covering ileostomy was constructed. However, complete elimination of the tumor appeared to be difficult due to multiple peritoneal metastases, spreading to the nodules, diaphragm and spleen.

**Figure 3:**
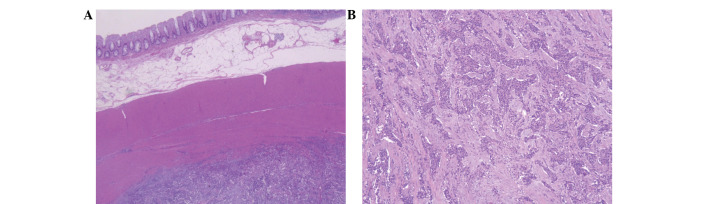
Microscopic findings of the resected specimen. (A) Tumor mass in the adventitial region of the rectum accompanied by invasive growth into the proper muscle layer (H&E; magnification, ×20). (B) Spindle-shaped tumor cells embedded in the abundant desmoplastic stroma (H&E, magnification, ×100).

**Figure 4:**
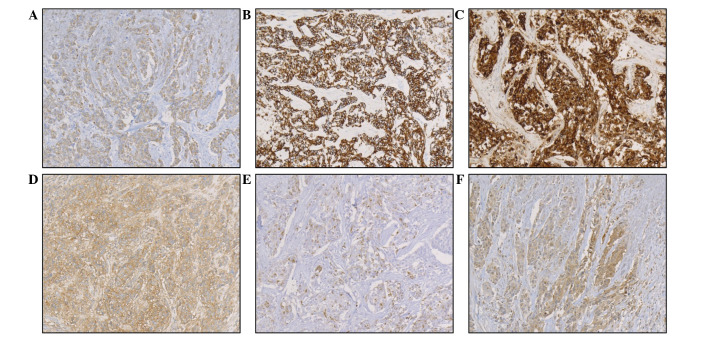
Immunohistochemical staining of the tumor spindle cells (magnification, ×100). Positive staining for (A) CK AE1/AE3; (B) CK CAM5.2; (C) EMA; (D) CD99; (E) desmin and (F) NSE were noted. CK, cytokeratin; EMA, epithelial membrane antigen; CD99, cluster of differentiation 99.

Macroscopically, the tumor, which measured 18×11×7 cm, had an uneven surface and when cut, necrotic areas were observed. Histological examinations of the excised tumor revealed that the tumor cells had focally invaded the muscularis propria of the rectum (Fig. 3A). Invasive small round or short spindle cells were identified embedded in the desmoplastic stroma (Fig. 3B). The tumor cells had round to oval nuclei with increased chromatins, which were accompanied by eosinophilic cytoplasm with numerous mitotic features. Foci of necrosis and vascular permeation were frequently observed. Immunohistochemical investigations revealed positive staining for cytokeratin (CK) AE1/AE3, CK CAM5.2, epithelial membrane antigen (EMA), cluster of differentiation (CD)99, desmin and NSE (Fig. 4), but negative staining for CK 34βE12, CK 5/6, CK 7, CK 20, c-kit, CD34, S-100 protein, CA 125, neurofilament, synaptophysin and α-smooth muscle actin (α-SMA). The MIB-1 index was 70% and focal positive staining for Wilms tumor-1 (WT1) was also noted. These immunohistological results supported the diagnosis of a progressing DSRCT. The postoperative period was uneventful and the patient was discharged 14 days later.

The patient was followed up and one year later, complained of intermittent pain in the right lower limbs. A follow-up examination by CT scan revealed metastasis and growth of tumor cells in the inguinal lymph nodes. A right inguinal lymphadenectomy was performed and five days later, the patient was discharged with no postoperative complications. The excised lymph nodes measured 4.5×3.0 and 5.0×2.0 cm. In the immunohistochemical examinations of the excised lymph nodes, CK AE1/AE3, CK CAM5.2, EMA, CD99, desmin, NSE, CK 7, c-kit and CA 125 were positive, however, CK 34βE12, CK 5/6, CK 20, CD34, S-100 protein, neurofilament, synaptophysin and α-SMA were negative. The MIB-1 index was 70% and these pathological findings of the lymph nodes were compatible with the metastatic DSRCT. At the present time, 60 months following the first diagnosis of DSRCT, the patient continues to be symptom free, without any local progression of the tumor.

## Discussion

DSRCT is a rare, but aggressive type of tumor with a poor prognosis and a high prevalence in young males, with the peak age at diagnosis ranging between 16 and 26 years (5,6). In the majority of cases, DSRCT appears as a large mass in the abdominal cavity with serosal and omental spreading, and rapidly metastasizes to the liver, lungs, lymph nodes and the peritoneum (7,8). The most common symptoms of DSRCT are non-specific. The majority of patients present with a palpable mass in the abdominal cavity, coupled with pelvic lesions, and the associated symptoms include abdominal pain, constipation, weight loss and distension (7). Accordingly, the patient in the present case complained of lower abdominal and pelvic pain with impaired bowel movement. Imaging techniques, including MRI and CT scans, are indispensible as tools for diagnosis, identification of tumor location and assessment of tumor progression. An intra-abdominal DSRCT often appears as multiple bulky, lobulated and heterogeneous masses, with hypodense areas in unenhanced CT images, and weak heterogeneous areas in contrast-enhanced CT images (5,9). Malignant peritoneal mesothelioma, rhabdomyosarcoma and lymphoma may demonstrate a radiographic appearance similar to DSRCT and should therefore be considered for differential diagnosis (10). In the present case, abdominal enhanced CT scan revealed that the large mass with weak heterogeneous enhanced areas in the pouch of Douglas, involved the rectosigmoid colon and a number of nodules (or seeding) in the abdominal cavity, in addition to metastasis to the inguinal lymph nodes. No calcification or heterogeneous hypodense areas were identified. In MRI examinations, DSRCT often presents as lesions with heterogeneous iso- or hypointense areas in T1-weighted MR images and heterogeneous hyperintense in T2-weighted MR images (10,11). In the present case, the large mass of the pouch of Douglas appeared hypointense in T1-weighted images and heterogeneous hyperintense in T2-weighted images.

DSRCT is a member of the family of malignant small round cell tumors. They are characterized by small, round, relatively undifferentiated cells and generally include Ewing’s sarcoma, peripheral neuroectodermal tumor, rhabdomyosarcoma, synovial sarcoma, non-Hodgkin’s lymphoma, retinoblastoma, neuroblastoma, hepatoblastoma, and nephroblastoma or Wilms tumor. Differential diagnosis of small round cell tumors is particularly difficult due to their undifferentiated or primitive features (12). Histopathologically, the majority of tumors exhibit a nesting or solid/diffuse pattern, with a characteristic histological appearance of high cellularity, undifferentiated small to medium size uniform round cells and a sparse cytoplasm. Nuclei are round to oval shaped and morphologically arranged in nest or spindle cell formation, embedded in dense and metachromatic desmoplastic stroma (13–15). The cells have high nuclear/cytoplasmic ratios with granular chromatin that is reminiscent of small cell carcinoma and pseudorosettes are observed in certain specimens. These features are key to the diagnosis of DSRCT. Currently, the diagnosis of DSRCT is based on immunohistochemical and molecular analysis, which are used as tools for the confirmation of diagnosis. Furthermore, DSRCT is a unique tumor with multiple phenotypic differentiations and characteristic immunohistochemical features. These features reflect diversity in the morphology of DSRCT, exhibiting characteristic features with regard to the epithelium, muscles and nerves. Previous studies have demonstrated that DSRCT exhibited strong and diffuse cytoplasmic immunoreactivity for CK AE1/AE3, vimentin, desmin, NSE and EMA, however, S-100 protein, chromogranin A, cynaptophysin and neurofilament were negative or weak (16–18). In the present case, positive immunoreactivity was observed for CK AE1/AE3, CK CAM5.2, EMA, CD99, desmin, NSE and WT1, but were negative for CK 34βE12, CK 5/6, CK 7, CK 20, c-kit, CD34, S-100 protein, CA 125, neurofilament, synaptophysin and α-SMA. The latter results were not consistent with a diagnosis of DSRCT.

The prognosis of patients with DSRCT remains poor and despite the availability of therapeutic strategies, including surgical resection combined with radiotherapy and chemotherapy, the mortality rates remain high. Although surgery, chemotherapy, radiotherapy and combined therapy have been used in the treatment of DSRCT, no single therapy has been accepted as the standard strategy. Complete excision is often difficult due to the presence of multiple or diffuse metastases in the peritoneum. In general, the patients with metastasis have a poor prognosis even following chemotherapy and/or radiotherapy. In a review of 66 patients with DSRCT, Lal *et al* (7) reported that the overall three and five-year survival rates were 44 and 15%, respectively. Gross tumor resection was highly significant in overall survival as the three-year survival rate in patients that were treated by gross tumor resection was 58%. Debulking surgery is attempted with a goal of ≥90% resection of the tumor bulk and aggressive surgical resection continues to be a major determinant of patient survival. Gil *et al* (17) reported that the median survival time of patients with complete cytoreduction was 20 months (range, 13–55 months). In another study, the 12 patients with DSRCT were reviewed and the median survival time of patients who underwent surgical resection compared with those who underwent biopsy alone was 34 versus 14 months, respectively (6). In 7/12 patients, surgical resection was attempted, however, macroscopic total resection of the tumor was accomplished in 3/7 patients and the remaining four patients underwent major debulking surgery. All patients who underwent macroscopic total resection subsequently developed recurrence, which required additional surgery.

Tumor recurrence and progression is common in patients with DSRCT. Accordingly, decisions regarding the appropriate follow-up treatment strategy are essential. Aggressive surgery combined with multi-agent adjuvant chemotherapy is recommended to relieve symptoms and to improve the outcome. Numerous aggressive combination chemotherapy protocols, including IRS-38 (oncovin, platinol, adriamycin, cyclophosphamide), VAC (oncovin, endoxan, actinomycin-D), IVA (ifosfamide, vincristine, adriamycin), P6 (cyclophosphamide, doxorubicin, vincristine, ifosfamide, etoposide) and PAVEP (cyclophosphamide, etoposide, doxorubicin, cisplatin), have been attempted with a certain degree of chemosensitivity and improved survival rates (19–22). However, during aggressive chemotherapy, drug toxicity may be severe and often requires hospitalization. Regarding a standard strategy for the treatment of DSRCT, neoadjuvant chemotherapy, >90% tumor debulking and radiotherapy have been demonstrated to prolong survival (4). Aggressive surgical resection of extensive intra-abdominal DSRCT correlates with an improvement of therapeutic outcomes (5). In the present case, the patient underwent surgical resection of a large abdominal tumor prior to admission to our hospital, however, the tumors had evidently not been removed completely because numerous nodules in the abdominal cavity were observed. Although chemotherapy had been applied in another hospital, the patient’s tumor was progressing upon our first examination. The patient refused additional chemotherapy and was followed up. One year later, the residual tumor had grown rapidly regardless of the surgery. To reduce the patient’s symptoms, debulking surgery was performed twice. At the time of writing, the patient continues to survive with no symptoms and the initial surgery was 60 months ago. Therefore, it was hypothesized that surgical debulking relieves symptoms and improves survival time in metastatic and recurrent DSRCT patients. Recent efforts have focused on improving disease control without increasing treatment-associated morbidity (23).

In conclusion, this case report presents a potential treatment strategy for patients who develop recurrence of intra-abdominal DSRCT as a solitary mass with multiple seeding of the peritoneum.
